# Wound healing potential of lavender oil by acceleration of granulation and wound contraction through induction of TGF-β in a rat model

**DOI:** 10.1186/s12906-016-1128-7

**Published:** 2016-05-26

**Authors:** Hiroko-Miyuki Mori, Hiroshi Kawanami, Hirohisa Kawahata, Motokuni Aoki

**Affiliations:** Graduate School of Health Sciences, Morinomiya University of Medical Sciences, 1-26-16, Nanko-kita, Suminoe-ku, Osaka 559-8611 Japan; Department of Chemistry, Wakayama Medical University, 580 Mikazura, Wakayama, 641-8509 Japan

**Keywords:** Complementary and alternative medicine, Lavender oil, Wound healing, TGF-β, Collagen

## Abstract

**Background:**

Although previous studies have suggested that lavender oil promote wound healing, no study has examined the molecular mechanisms of its effect. In this study, we investigated the effect of lavender oil on various steps of wound healing and its molecular mechanism, focusing on transforming growth factor-β (TGF-β).

**Methods:**

Circular full-thickness skin wounds were produced on rats. Control solution or lavender oil was topically applied to the wounds on alternating days for 14 days.

**Results:**

The area of wounds topically treated with lavender oil was significantly decreased as compared to that of wounds of control rats at 4, 6, 8, and 10 days after wounding. Topical application of lavender oil induced expression of type I and III collagen at 4 days after wounding, accompanied by an increased number of fibroblasts, which synthesize collagen. Induced expression of type III collagen by topical application of lavender oil was reduced to control level at 7 days after wounding although increased expression of type I collagen still continued even at 7 days, suggesting rapid collagen replacement from type III to type I in wounds treated with lavender oil. Importantly, expression of TGF-β in wounds treated with lavender oil was significantly increased as compared to control. Moreover, an increased number of myofibroblasts was observed in wounds treated with lavender oil at 4 days after wounding, suggesting promotion of differentiation of fibroblasts through induction of TGF-β, which is needed for wound contraction.

**Conclusion:**

This study demonstrated that topical application of lavender oil promoted collagen synthesis and differentiation of fibroblasts, accompanied by up-regulation of TGF-β. These data suggest that lavender oil has the potential to promote wound healing in the early phase by acceleration of formation of granulation tissue, tissue remodeling by collagen replacement and wound contraction through up-regulation of TGF-β. The beneficial effect of lavender oil on wound healing may raise the possibility of new approaches as complementary treatment besides conventional therapy.

## Background

The use of complementary and alternative medicines (CAMs) to treat a variety of conditions is increasing, and interest in their potential has been growing all over the world. Aromatherapy, which employs essential oils extracted from various plants and herbs, is widely used and is becoming a major CAM. Among various CAMs, inhalation aromatherapy has especially received attention mainly for its effects of relaxation and improvement of emotional or psychological conditions, and some clinical trials have suggested the potential of aromatherapy for anxiety [1], insomnia [2], stress [3], and pain [4]. Regarding anxiety, efficacy on the clinical outcome is partially supported by a few studies demonstrating an anti-conflict effect of essential oils [5, 6] and a metabolic response to inhalation of essential oils in anxiety model rats [7]. However, despite the reported beneficial effect in such clinical trials, these effects are still controversial because of lack of adequate basic experiments, the small number of recruited subjects, and lack of rigorous analytical methods. Further studies, especially basic studies to elucidate the detail mechanisms of the effects of essential oils, are needed.

In such situations, the researchers’ interest in the biological and physiological activities of essential oils has been increased. In in vitro and in vivo experiments, some essential oils were suggested to act as anti-inflammatory [8], anti-viral [9], anti-tumor [10], anti-hyperglycemic [11], and anti-carcinogenic [12] agents. In response to these results, targets of aromatherapy and its therapeutic potentials have expanded from emotional and psychological symptoms to various physical diseases. Moreover, these reports unexpectedly suggest that aromatherapy could be effective through not only inhalation, but also topical application of essential oils, and the medical indications of aromatherapy have expanded. Wound healing is one of the expected targets of topical application of essential oils. This has arisen because a more efficient strategy is still required in cases of unsuccessful or deficient repair. Useful CAMs that offer an easy approach, less toxicity and fewer side-effects in combination with conventional therapy are anticipated for severe ischemic ulcers or bedsores that are intractable due to insufficient growth of granulation tissue and lack of blood supply. It is already reported that some essential oils promote wound healing [13–17].

Lavender essential oil is expected to have a beneficial effect on wound healing because a few evidences for its effect were already reported [16–21]. A previous randomized control trial conducted on 120 women demonstrated that treatment with lavender oil significantly reduced pain after episiotomy and redness of incision sites as compared to control [18]. More recently, another randomized clinical trial for episiotomy demonstrated the similar results; significant reduction of REEDA (redness, edema, ecchymosis, discharge and approximation) score and visual analogue scale score for pain, as compared to control [19]. Both clinical trials suggest beneficial effect of lavender oil on wound healing. Also, it was reported that topical treatment with lavender oil on aphthous ulceration showed a significant ulcer size reduction as compared to control in both an animal experiment and a clinical study [20]. Moreover, there is a report evaluating the mechanism of effect of lavender oil on cutaneous wound healing in an animal experiment [21]. This paper demonstrated that wound closure progressed more rapidly with topical application of lavender oil as compared to the control, accompanied by increased expression of PDGF-A and EGF, which are growth factors playing important roles in wound healing process such as tissue remodeling and re-epithelialization [21].

These clinical trials and animal experiments strongly suggest wound healing potential of lavender oil. However, elucidation of the mechanisms, especially the molecular mechanism, is not enough, and it is still unclear how essential oils act on various parts of the wound healing process. Thus, in this study we investigated effect of lavender oil on wound healing and its molecular mechanism, using a cutaneous wound animal model. As wound healing process consists of sequential events such as formation of granulation tissue, collagen replacement from type III to type I and wound contraction (wound shrinking), we evaluated the influence of lavender oil on each part of wound healing in this study. Moreover, expression of transforming growth factor-β (TGF-β) was evaluated as a key molecule playing a role in healing of wounds topically treated with lavender oil, because TGF-β is known to regulate proliferation of fibroblasts, collagen synthesis in fibroblasts, production of wound granulation tissue [22], and differentiation of fibroblasts to myofibroblasts in granulation tissue [23]. Here, we demonstrated the wound healing potential of lavender oil through induction of TGF-β in an animal model.

## Methods

### Procedure of animal experiment

Male Sprague-Dawley rats obtained from Japan SLC (Shizuoka, Japan), weighing about 250–270 g, were used in this study. The rats were maintained under constant room temperature (20–25 °C) with free access to water and a standard diet throughout the study.

The rats were anesthetized with 1.5 % halothane using an induction chamber and intraperitoneal administration of pentobarbital (0.5 ml/kg). After shaving the hair on their back and cleaning with 70 % ethanol, a circular full-thickness skin wound (10 mm in diameter) was made in the midline of the back of each animal. Lavender oil (*Lavandula angustifolia* 0.8896 g/ml density) was dissolved up to 1 % in solution containing 0.1 % DMSO and Tween 20 because of its lipophilicity. Rats were randomly divided into three groups: (1) Untreated group; wound surgery only, (2) Control group; wound topically treated with control solution containing 0.1 % DMSO and Tween 20, and (3) Lavender group; wound topically treated with 1 % lavender oil dissolved in control solution. Then, 50 μl of each solution was applied to the wound area just after wound surgery, and each treatment was continued on alternating days till 14 days after surgery. As application of diluted essential oils to the skin or a wound is a popular approach in humans [24], diluted lavender essential oil (1 % solution) was applied to wounds without any ointment base or oleaginous base, in order to avoid the additional effect of these bases on the wound healing process. Each rat was separated to prevent licking the solution and to avoid serious infection of the wound. The wound area was digitally photographed at 0, 2, 4, 6, 8, 10, 12 and 14 days after wound surgery using a digital camera (Canon Power Shot S200, Tokyo, Japan), then the area was quantified using an image analysis system, Image J (NIH). Measurements were performed in a blind manner. Each investigator was blinded to group assignment and other data concerning the animals, as well as to the results of the other investigator. Rats were sacrificed by intraperitoneal administration of an overdose of pentobarbitonein, to isolate tissue samples from skin for investigations.

### Chemicals and reagents

Lavender oil was purchased from Pranarom, Int. (Ghislenghien, Belgium). Details about the chemical composition of lavender oil are shown in Table 1. The lavender oil we used was extracted by the hydrodistillation method from Lavandula angstiforia ssp. angstiforia. It was a pure essential oil, and no other substances including ointment base were added to the distilled extract, in order to exclude the effect of other components. Tween 20 and DMSO were purchased from Wako Pure Chemical Industries (Osaka, Japan) and Sigma-Aldrich Co. (St Louis, MO, USA), respectively. All other chemicals were analytical grade.

**Table 1:** Details about the chemical composition of lavender oil

Constituent	%	Constituent	%
Monoterpene alcohols	47.52 %	Monoterpene hydrocarbons	5.09 %
linalool	43.00 %	trans-β-ocimene	1.92 %
borneol	1.80 %	cis-β-ocimene	1.47 %
α- terpineol	1.02 %	camphene	0.28 %
terpinen-4-ol	0.91 %	limonene	0.25 %
geraniol	0.59 %	others	
others			
Esters	34.81 %	Sesquiterpene hydrocarbons	4.58 %
linalyl acetate	32.09 %	β-caryophyllene	2.80 %
lavandulyl acetate	1.29 %	β-farnesene	1.46 %
1-octen-3-yl acetate	0.59 %	germacrene D	0.15 %
hexyl acetate	0.40 %	others	
others		Ketones	2.05 %
		3-octanone	1.22 %
		Camphor	0.83 %
		others	

### Immunohistochemical studies

Rats were sacrificed at 4 days after wounding, and skin tissue was isolated from the wound lesion for histological examination. Skin tissues were fixed in 10 % formalin for 24 h and processed for routine paraffin embedding.

For immunohistochemical staining, anti-type III collagen (Col III) antibody, antibody to α-smooth muscle actin (α-SMA) (Abcam, Cambridge, MA, USA) and monoclonal antibody to prolyl-4-hydroxylase (P4H) (Acris Antibodies, Inc., CA, USA) were used to analyze the expression of type III collagen and collagen synthesis in fibroblasts, respectively, in wound lesions of the rat model. Also, antibody to α-SMA was used to detect myofibroblasts. Immunohistochemical staining was performed using the high polymer, HISTOFINE simple stain rat MAX-PO (Nichirei Bioscience Inc., Tokyo, Japan) method. Then, 5-μm sections were deparaffinized, rehydrated before blocking endogenous peroxidase activity with 3 % hydrogen peroxide, and pre-incubated with 1.5 % blocking reagent (Roche Applied Science, Indianapolis, USA) in Tris-HC1 buffered saline (TBS) for 1 h. Diluted primary antibodies (Col III 1:600, P4H 1:20, α-SMA 1:100) were then applied to the sections, and these sections were incubated for 1 h. Following this, the specimens were rinsed twice with TBS for 5 min and incubated with HISTOFINE simple stain rat MAX-PO (mouse) (Nichirei Bioscience Inc.) for 30 min. Peroxidase activity was visualized by treatment with 0.05 % diaminobenzidine containing 0.3 % hydrogen peroxide. After the samples were rinsed in water, the sections were dehydrated, cleared and mounted.

### Expression of mRNA of type I collagen and type III collagen

Rats were sacrificed at 4 and 7 days after wounding, and skin tissue was isolated from wound lesions. Excised tissues were homogenized in cold PBS and centrifuged at 20,000 g for 15 min at 4 °C. Total RNA of tissue samples was extracted using ISOGEN II (NIPPON GENE, Toyama, Japan) and re-suspended in PBS, and purity was assessed by spectrophotometry. Only samples with a ratio of spectrophotometric absorbance at 260 nm to that at 280 nm (A260/A280) in the range of 1.9–2.1 were used. Complementary DNA was synthesized using an iScript cDNA Synthesis Kit (Bio-Rad Laboratories, Hercules, CA, USA). Amplification reactions were performed with SsoFast EvaGreen Supermix (Bio-Rad) with 1 μm of primers and 4 μl of cDNA in a final volume of 50 μl, and were carried out in a MiniOpticon Real-Time PCR Detection System (Bio-Rad), according to the manufacturer’s instructions (1 min at 95 °C followed by 40 cycles of 1 s at 95 °C and 5 s at 60 °C). The expression level of GAPDH as a housekeeping gene was used as the internal control, and the comparative Ct method (2^ΔΔCt^) was used to quantify gene expression levels. The values are shown as relative expression by delta Ct (subtraction of the crossing point cycle for the housekeeping gene from that of the gene analyzed). The oligonucleotides were synthesized by Gene Design (Suita, Japan). Sequences of primers are shown in Table 2 [25–27].

**Table 2:** Primers designed for real-time PCR

Name	Sense primer (5′-3′)	Antisense primer (5′-3′)
rat type I collagen a2	GCTTTGTGGATACGCGAACTC	CCAGCATTGGCATGTTGCT
rat type III collagen a1	TGATGGGATCCAATGAGGGAG	GAGTCTCATGGCCTTGCGTGTTT
rat GAPDH	ATGGCACAGTCAAGGCTGAGA	CGCTCCTG GAAGATGGTGAT

### Enzyme-linked immunosorbent assay for TGF-β

Rats were sacrificed at 4 and 7 days after wounding, and skin tissue was isolated from wound lesions to extract proteins in each group. Tissues were homogenized, and extraction of proteins was performed using T-PER Tissue Protein Extraction Reagent (Pierce, Rockford, IL, USA) according to the manufacturer’s instructions. Protein concentrations were determined using Bio-Rad Protein Assay (Bio-Rad, Hercules, CA) reagent with bovine serum albumin as standard. Protein levels of TGF-β in skin tissue were measured using an enzyme-linked immunosorbent assay (ELISA) kit according to the manufacturer’s instructions (RayBiotech Inc., Norcross, GA, USA).

### Statistical analysis

All numerical values are expressed as mean ± SEM. Data sets were analyzed by unpaired Student’s t-test or analysis of variance (ANOVA) followed by Tukey-Kramer post hoc test. Differences with *p* <0.05 were considered statistically significant.

## Results

### Effect of lavender oil on wound healing

**Fig. 1:**
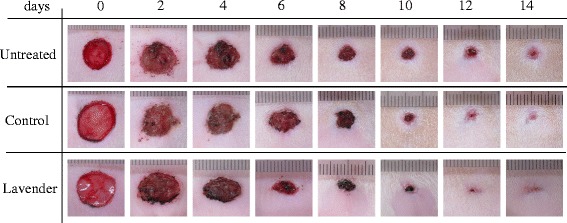
Representative photographs of transition of wound closure in rat model. Untreated; wound surgery only, Control; wound topically treated with control solution containing 0.1 % DMSO and Tween 20, Lavender; wound topically treated with 1 % lavender oil dissolved in control solution

**Fig. 2:**
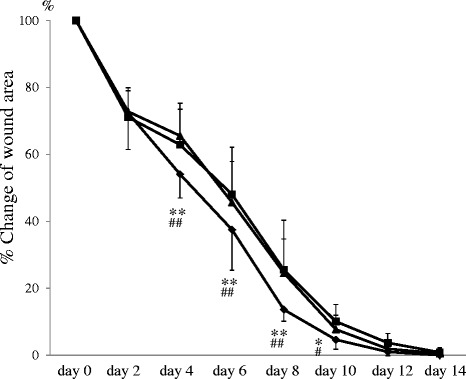
Transition of wound area measured by Image J. Untreated (▲); rats with surgery only, Control (■); rats treated with control solution containing 0.1 % DMSO and Tween 20, Lavender (◆); rats treated with 1 % lavender oil dissolved in control solution. Values are mean ± SEM. *n* = 6 in each group. **: *P* <0.01 vs untreated, *: *P* <0.05 vs untreated, ##: *P* <0.01 vs Sham, #: *P* <0.05 vs Sham

Representative photographs of the process of wound healing in each group are shown in Fig. 1. The photographs suggest that topical application of lavender oil promotes wound closure, with a reduction in wound area. To perform accurate and quantitative analysis, the area of wound lesions in each group was measured using Image J at 0, 2, 4, 6, 8, 10, 12, and 14 days after wounding (Fig. 2). There was no significant difference in the wound area between untreated and control rats at each time point. However, wound closure was observed to progress more rapidly with topical application of lavender oil. The wound area of rats treated with lavender oil was significantly decreased as compared to that of untreated rats and control rats at 4, 6, 8, and 10 days after wounding (at day 4, 6, 8: *p* <0.01 vs untreated and control; at day 10: *p* <0.05 vs untreated and control) (Fig. 2). There was no significant difference in wound size at 12 and 14 days (Fig. 2). These data suggest wound healing potential of lavender oil in the early phase. Serious infection was not observed in each animal of each group, and it was considered that there was little effect of infection on wound healing in our experiment.

### Proliferation of fibroblasts and synthesis of collagen

**Fig. 3:**
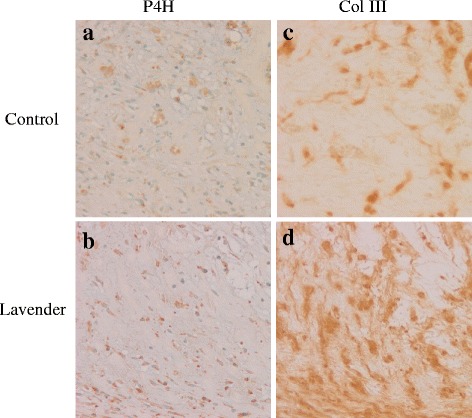
Representative photomicrographs of immunohistochemical studies. **a**, **b** Immunohistochemical staining for P4H at 4 days after wounding. **c**, **d** Immunohistochemical staining for type III collagen at 4 days after wounding. Magnification; × 100. Control; wound topically treated with control solution containing 0.1 % DMSO and Tween 20, Lavender; wound topically treated with 1 % lavender oil dissolved in control solution

Immunohistochemical studies demonstrated an increased number of P4H-positive cells, indicating fibroblasts that synthesize collagen, in wound lesions topically treated with lavender oil as compared to that in wound lesions treated with control solution (Fig. 3a, b). Then, we assessed collagen secretion by fibroblasts. As shown in Fig. 3c and d, immunochemical staining showed that production of type III collagen (Col III), which is essential for formation of granulation tissue in the early phase of wound healing, was increased by topical treatment with lavender oil, accompanied by an increased number of P4H-positive fibroblasts (Fig. 3b).

**Fig. 4:**
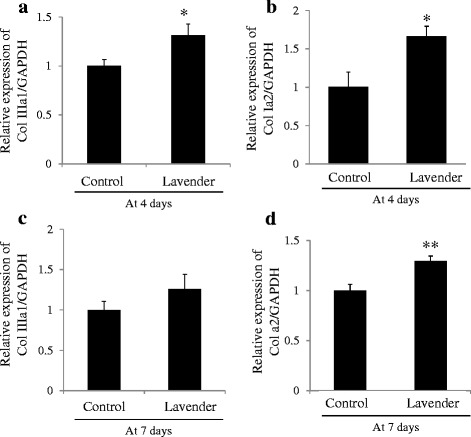
Expression of mRNA of type I collagen and type III collagen. **a** Relative mRNA expression of type III collagen (Col IIIa1) at 4 days after wounding. **b** Relative mRNA expression of type I collagen (Col Ia2) at 4 days after wounding. **c** Relative mRNA expression of type III collagen (Col IIIa1) at 7 days after wounding. **d** Relative mRNA expression of type I collagen (Col Ia2) at 7 days after wounding. Control; wound topically treated with control solution containing 0.1 % DMSO and Tween 20, Lavender; wound topically treated with 1 % lavender oil dissolved in control solution. Values are mean ± SEM. *n* = 6 in each group. *: *p* <0.05 vs Control, **: *p* <0.01 vs Control

Also, expression of each collagen was confirmed by RT-PCR. At 4 days after wounding, expression of mRNA of both type III collagen (Col IIIa1) and type I collagen (Col Ia2) in skin tissues of wound lesions topically treated with lavender oil was significantly increased as compared to that of those treated with control solution (*p* <0.01 vs control) (Fig. 4a, b), suggesting acceleration of formation of granulation tissue in the early phase of wound healing. Moreover, expression of Col IIIa1 in wound lesions topically treated with lavender oil rapidly decreased to the control level by 7 days after wounding (Fig. 4c), while significantly higher expression of Col Ia2 by treatment with lavender oil as compared to control was still observed even at 7 days after wounding (Fig. 4d). This suggests that treatment with lavender oil results in promotion of tissue remodeling by rapid replacement of type III collagen with type I collagen. There was no significant difference in expression of Col Ia2 and Col IIIa1 between the untreated group and control group (4 days; Col Ia2/GAPDH of untreated: 0.93 ± 0.19, Col IIIa1/GAPDH of untreated: 1.04 ± 0.16, ns vs that of Control, respectively) (7 days; Col Ia2/GAPDH of untreated: 0.93 ± 0.19, Col IIIa1/GAPDH of untreated: 1.08 ± 0.08, ns vs that of Control, respectively).

### Expression of TGF-β and differentiation of fibroblasts to myofibroblasts

**Fig. 5:**
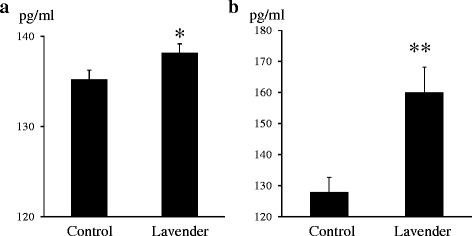
Expression of TGF-β protein determined by ELISA. **a** Expression of TGF-β protein at 4 days after wounding. **b** Expression of TGF-β protein at 7 days after wounding. Control; wound topically treated with control solution containing 0.1 % DMSO and Tween 20, Lavender; wound topically treated with 1 % lavender oil dissolved in control solution. Values are mean ± SEM. *: *p* <0.05 vs Control, **: *p* <0.01 vs Control. *n* = 6 in each group

To investigate the detailed molecular mechanism of the effect of lavender oil on wound healing, we focused on expression of TGF-β, as it is already known to induce proliferation of fibroblasts and synthesis of both type I and type III collagen [22]. Interestingly, as shown in Fig. 5, our ELISA study demonstrated that the protein level of TGF-β in wound lesions topically treated with lavender oil was significantly increased as compared to that in those treated with control solution, at both 4 and 7 days after wounding (at day 4, *p* <0.05 vs control, at day 7: *p* <0.01 vs control). There was no significant difference in expression of TGF-β between the untreated group and control group (4 days; untreated: 134.73 ± 3.94 pg/ml, Control: 136.44 ± 12.91 pg/ml, ns. 7 days; untreated: 134.17 ± 1.9 pg/ml, Control: 130.97 ± 4.89 pg/ml, ns).

**Fig. 6:**
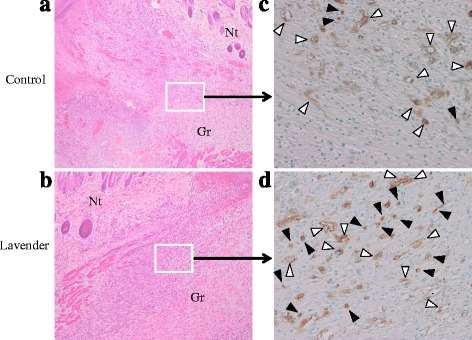
Representative photomicrographs of immunohistochemical studies. **a**, **b** Wound lesion stained by hematoxylin-eosin staining at 4 days after wounding. Magnification; × 40. **c**, **d** Immunohistochemical staining for α-SMA at 4 days after wounding. Magnification; × 200. Control; wound topically treated with control solution containing 0.1 % DMSO and Tween 20, Lavender; wound topically treated with 1 % lavender oil dissolved in control solution. *Gr* granulation, *Nt* normal tissue. Closed arrow heads indicate representative myofibroblasts and open arrow heads indicate representative vascular smooth muscle cells around vasculature

In addition, TGF-β was reported to promote differentiation of fibroblasts into myofibroblasts in wound granulation tissue [23]. Differentiation to myofibroblasts in wound lesions is essential for wound contraction. Consistent with the previous report, an increased number of myofibroblasts positively stained for α-SMA (open arrow heads) was observed in wound lesions topically treated with lavender oil at 4 days after wounding, in the early phase of the wound healing process (Fig. 6). This suggests that treatment with lavender oil accelerates wound contraction by myofibroblasts.

## Discussion

Wound healing is a natural physiological process that develops in response to tissue damage, to restore the function and integrity of damaged skin tissues. The wound healing process is divided into four overlapping phases; blood clotting, inflammation, new tissue formation, and tissue remodeling [28]. These processes, especially new tissue formation and tissue remodeling, consist of sequential and coordinated events including angiogenesis, cellular proliferation, collagen synthesis followed by formation of granulation tissue, matrix degradation followed by replacement of collagen, wound contraction, and scar formation [29–32]. These healing processes are regulated by a large number of growth factors, cytokines, mitogens and chemotactic factors. Among them, epidermal growth factors (EGFs), insulin-like growth factors (IGFs), platelet-derived growth factors (PDGFs), and fibroblast growth factors (FGFs) are considered to play an important role in wound healing because these growth factors regulate cell migration and proliferation and the synthesis of extracellular matrix proteins, which are essential for formation of granulation tissue [22, 33, 34]. Indeed, there have been several studies of the transfer into wounds of some of these genes to promote wound healing and accelerate wound repair [35–37]. Besides these growth factors, there has been a focus on TGF-β in the wound healing process as it is considered to have the broadest spectrum of effects [22, 38, 39].

Lavender oil is extracted from *Lavandula angstiforia ssp. angstiforia* and is popularly used as a CAM in various fields of health promotion. There are many reports suggesting beneficial effects of inhalation of lavender oil on pain [40], allergic airway inflammation of asthma [41], anxiety disorder [42], quality of sleep [2, 43], and dementia [44]. Besides these expected effects, the influence of topical application of lavender oil on wound healing has already been evaluated [16–21]. Although previous studies suggested a beneficial effect of lavender oil on wound healing, the detailed mechanisms of the effect have not been fully elucidated. However, there is an interesting paper demonstrating that wound closure progressed more rapidly with topical application of lavender oil as compared to the control, and that expression of PDGF-A and EGF tended to increase, although there was no significant difference as compared to the control [21]. As PDGF-A is known to induce the secretion of matrix metalloproteinases (MMPs) from fibroblasts, this study suggests that lavender oil may accelerate wound closure through a rapid decrease in granulation tissue induced by PDGF and progression of re-epithelialization induced by EGF. To our knowledge, this was the only study to refer and suggest molecular mechanisms of the effect of lavender oil on wound healing.

In contrast, in the present study, we demonstrated that acceleration of the formation of granulation tissue in the early phase, rather than a rapid decrease in granulation as previously reported [21], leads to rapid remodeling by collagen replacement and promotes wound closure. The current study showed that topical treatment with lavender oil increased the number of fibroblasts positively stained for P4H, which catalyzes proline hydroxylation of procollagen and is essential for collagen maturation and synthesis in fibroblasts [45, 46], and induced expression of both type I and III collagen in wound lesions. The obtained data suggest that topical treatment with lavender oil accelerates formation of granulation tissue in the early phase of wound healing. Moreover, topical application of lavender oil to wounds is suggested to advance collagen replacement from type III to type I, based on our findings that increased expression of type I collagen was observed even at 7 days, although expression of type III collagen rapidly decreased to the control level by 7 days. As formation of granulation tissue consisting of collagen and replacement of type III collagen with type I collagen are essential for tissue remodeling in the wound healing process, acceleration of formation of granulation and collagen replacement in the early phase is considered to promote wound healing. In fact, the wound area of rats treated with lavender oil was significantly decreased as compared to that of untreated rats and control rats at 4, 6, 8, and 10 days after wounding.

A novel finding of the current study is the induction of expression of TGF-β by treatment with lavender oil. There has been no report of a complication of TGF-β induction in the wound healing activity of essential oils. It has been reported that TGF-β stimulates angiogenesis, proliferation of fibroblasts, and matrix production by fibroblasts [22, 47, 48], and that it could be one of key molecules in the wound healing process [32, 37, 38, 49]. From these previous reports, TGF-β is considered to play a prominent role in cutaneous wound healing by acceleration of formation of granulation tissue, accompanied by increased production of collagen by fibroblasts. Thus, the up-regulation of TGF-β by treatment with lavender oil observed in the present study can rationally explain the proliferation of fibroblasts which synthesize collagen and increased mRNA expression of collagen. TGF-β and collagen are considered to be expressed in a coordinated manner to form granulation in the wound. Also, TGF-β was reported to induce secretion of MMP-13, so-called collagenase-3, by fibroblasts [50]. MMP-13 is essential for degradation of type III collagen, followed by replacement by type I collagen and tissue remodeling in the process of wound healing. In the present study, despite a significant increase in type III collagen at 4 days after the start of topical treatment with lavender oil, it rapidly decreased to the control level by 7 days. Rapid degradation of type III collagen is strongly suggested to be mediated by MMP-13, which is induced by up-regulation of TGF-β. Taken together, these findings indicate that induction of TGF-β by lavender oil functions to accelerate not only the formation of granulation tissue but also the replacement of collagen.

Another important finding of the present study is that topical treatment with lavender oil promoted differentiation of fibroblasts to myofibroblasts in wound granulation in the early phase of wound healing. This can also be explained by up-regulation of TGF-β, because TGF-β has been reported to stimulate differentiation of fibroblasts to myofibroblasts [23], which play a major role in tissue shrinking/contraction in the wound healing process. Also, it was reported that stimulation of the TGF-β signaling pathway by angiotensin II induces granulation tissue contraction via the angiotensin type 1 receptor [51]. Thus, the present data also showed that induction of TGF-β by topical application of lavender oil promotes not only formation of granulation tissue, but also wound shrinking/contraction. Moreover, a previous study demonstrated that suppression of type III collagen in the wound area enhanced myofibroblast expression [52], and that diminished type III collagen promotes myofibroblast differentiation [49]. From this point of view, lavender oil may promote wound shrinking/contraction, because our data demonstrated that rapid degradation of type III collagen was observed in wounds topically treated with lavender oil.

There are some issues and study limitations of our study. First, it is unclear whether essential oils induce inflammation or not. The up-regulation of TGF-β observed in our study suggests that inflammation is induced in wound lesions topically treated with lavender oil, because inflammation is a major factor that induces the expression of TGF-β. Considering that inflammation is essential for progression of wound healing, our findings are rational. However, lavender is generally reported to be an effective medicinal plant for treating inflammation. Indeed, some papers suggest an anti-inflammatory effect of lavender oil [41, 53]. On the other hand, there is a report showing that other essential oils induce a cutaneous inflammatory response in wound lesions [54]. No conclusion has been reached because of insufficient basic studies. Further study is needed to resolve this question and to prove the rationality of the data obtained from this study. The second issue is that in the present study there was no difference in the total number of days (14 days) needed for complete healing, although the area of wounds topically treated with lavender oil was significantly decreased at 4, 6, 8, and 10 days after wounding. Our data indicate that lavender oil accelerates wound healing only in the early phase. This may be explained by lavender oil-induced promotion of formation of granulation tissue, replacement of collagen and wound contraction, which mainly occur in the early phase of wound healing. Especially, the decreased wound size after 4 to 10 days of treatment with lavender oil may be consequent upon wound contraction. On the other hand, considering the lack of difference in total days needed for complete healing, lavender oil may have no influence on re-epithelialization for wound closure. This is the opposite result to that of a previous study that suggested acceleration of re-epithelialization through induction of EGF by topical application of lavender oil [21]. Detailed investigation of the effect of lavender oil on expression of other growth factors and its influence on other steps of wound healing are awaited. Finally, it is still unclear which component of lavender oil (Table 1) has the observed effect in this study. Although linalool and linalyl acetate, which are the major components of lavender oil (Table 1), are reported to have an anti-microbial [55] and an anti-inflammatory effect [56], the participation of these components in the proposed mechanism of lavender oil was not evaluated in this study. As the aim of this study was to evaluate the effect of whole lavender oil which is popularly and practically used in humans, component analysis of the lavender oil we used and investigation of the effect of each constituent should be studied in further experiments.

## Conclusions

The present study demonstrated that topical treatment with lavender oil significantly increased collagen synthesis by fibroblasts, accompanied by enhanced expression of TGF-β in wound lesions. Also, rapid replacement of type III collagen with type I collagen in wounds treated with lavender oil was suggested by the finding that increased expression of type III collagen decreased to the control level by 7 days, while type I collagen was not reduced even at 7 days. Moreover, an increased number of myofibroblasts, probably due to up-regulation of TGF-β, was observed in the early phase of the wound healing process in wound lesions topically treated with lavender oil, suggesting that treatment with lavender oil also accelerates wound contraction by myofibroblasts. Overall, the present data demonstrated that topical application of lavender oil to wounds accelerates wound healing through 1) formation of granulation tissue by collagen synthesis, 2) tissue remodeling by collagen replacement from type III to type I, and 3) wound contraction (wound shrinking). Of importance, this paper firstly suggests that TGF-β is involved in the mechanism of the effect of lavender oil on wound healing. The beneficial effect of lavender oil on wound healing may raise the possibility of new approaches as complementary treatment besides conventional therapy.

## Abbreviations

α-SMA, α-smooth muscle actin; TGF-β, transforming growth factor-β; RT-PCR, real time polymerase chain reaction; P4H, prolyl-4-hydroxylase; CAM, complementary and alternative medicine
